# Enhanced Non-Contact Grip Force and Swirl Stability by a Combined Venturi–Vortex Air Head

**DOI:** 10.3390/ma14237123

**Published:** 2021-11-23

**Authors:** Yung Hoon Lee, Joon Hyun Kim, Jaeyong Sung

**Affiliations:** 1 Graduate School, Department of Mechanical Engineering, Seoul National University of Science &Technology, 232 Gongneung-ro, Nowon-gu, Seoul 01811, Korea; yun19997200@hanmail.net; 2 NDT Research Center, Seoul National University of Science &Technology, 232 Gongneung-ro, Nowon-gu, Seoul 01811, Korea; joonk61@seoultech.ac.kr; 3 Department of Mechanical and Automotive Engineering, Seoul National University of Science &Technology, 232 Gongneung-ro, Nowon-gu, Seoul 01811, Korea

**Keywords:** non-contact gripper, airhead, grip force, swirling flow, vacuum, venturi nozzle

## Abstract

A combination of the venturi module and the vortex cup was proposed to solve vortex instability and to enhance grip capacity. Mounting a venturi suction pad inside the vortex cup improved vacuum generation efficiency. When the vortex cup properly maintained the non-contact air gap and generated an equivalent vacuum to achieve a sealing effect around the open gap of the suction pad, the combined head improved grip capacity and stabilized the non-contact environment. Furthermore, the flow patterns around the venturi chamber and the swirl inside the vortex cup were analyzed based on the design elements of each module. In a module that integrated some of the venturi’s features internally, increased air consumption of the vortex cup was required than that of the venturi. However, it supported a wide range of non-contact grips. The coupled model effectively protected the vacuum suction features of the venturi suction pad in all non-contact environments in that range.

## 1. Introduction

Pneumatic technologies, which control fluid motion and apply it to transport systems, have been increasingly used in semiconductors, displays, solar panels, pharmaceuticals, and the food sector. Depending on the transport method, they require high precision and cleanliness. Although these technologies heavily rely on manufacturing skills, some options allow us to choose approaches in detail. The most common method is a simple system placed on a moving device, such as a conveyor or robot hand [1]. However, this traditional contact method is prone to surface scratches, static electricity, and damages in the working environment. Thus, non-contact methods have been developed to improve production efficiency [2,3]. Today, workpieces such as semiconductor wafers are gripped and managed by various non-contact airheads of robot handlers [4,5]. Moreover, air floating technology, which blows floor air and floats horizontally, is applied to plates, such as LCD and OLED panels [6].

The most widely used non-contact gripper works based on obtaining a grip force by controlling compressed air flows and simultaneously generating thrust and a suction force based on the Bernoulli principle [1,6,7,8,9,10], which ensures efficiently holding small, flat, and rigid workpieces in one direction. Conversely, if the workpiece is thin, heavy, and uneven, it is impossible to properly manage the grip environment at the end of the device by applying the Bernoulli principle. Current robotic applications require responding to non-contact curved surfaces with high grip performance. Thus, many ideas have been suggested for the head’s end-effector, but they have remained limited to small and hard objects [11].

The Bernoulli, venturi, vortex, and Coanda principles generate vacuum and adequate grips in traditional contact methods based on compressed air. However, it is challenging to obtain a strong vacuum capacity by increasing the head size or airflow rate in non-contact methods, let alone controlling airflow in open spaces for non-contact grip. Currently, non-contact head modules based on the Bernoulli or vortex principles are commercially available in diverse types and specifications, depending on the size and shape of the workpiece, material properties, or working conditions. In general, with a body diameter of up to 100 mm at 258 L/min air consumption, commercial vortex modules can provide a grip force of 44 N. The Bernoulli module provides a grip force up to 14 N for a maximum outer diameter of 150 mm at 291 L/min air consumption instead of providing smooth gripping operation [12]. As the maximum grip force that can be achieved in a non-contact environment is limited due to the availability of only one module specification, researchers have focused on finding the optimal shape design that allows more control over the flow behavior inside the module [13,14,15,16,17,18,19,20]. Their results significantly improved the air head’s internal structure and created an efficient vacuum on the workpiece.

This study proposes a non-contact gripper by adopting a combined venturi–vortex airhead to overcome limited grip force and flow instability in the non-contact operation mode. The combined module was reconstructed based on previous findings [21], which dealt with vortex cups and compact venturi nozzles [12]. Consequently, its head performance was numerically simulated. Based on the results, grip capacity under the non-contact environment and the effective condition of the head’s end are discussed.

## 2. Combined Venturi–Vortex Air Head

Various commercially applicable methods have been developed depending on the grip or hold type for process transportation in the industry [4,7,21]. Unidirectional non-contact grips are mostly preferred in compact devices by using the Bernoulli or vortex principles. The grip force must first be obtained in a vacuum; then, the non-contact gap is obtained by thrust acting in the opposite direction. Vacuum units suitable for this purpose include Bernoulli and vortex modules. Other vacuum units that use high-speed airflow without creating a non-contact environment include venturi and Coanda modules [22,23,24]. By using a combination of vacuum units with distinct characteristics, it is possible to simultaneously improve the non-contact environment and expand the range of applications. Several studies on the simultaneous use of two or more vacuum devices have been demonstrated to create a non-contact environment [18,25,26,27].

The presentative hybrid concept consists of the venturi nozzle, the venturi tube, and the Bernoulli nozzle, as shown in Ref. [26]. The venturi vent is a compression outlet, which serves as a Bernoulli nozzle and efficiently uses the compressed air’s kinetic energy. Suppose that the vortex principle is applied instead of the Bernoulli principle. In that case, the vortex flow can be induced by directing the exhaust port of the venturi nozzle toward the internal tangential wall. If the entire venturi is present inside the Bernoulli cup, it can have an extremely complicated structure [26]. Above all, the venting space of the venturi and the mechanisms of vacuuming by Bernoulli’s airflow behavior have not been clearly established.

**Figure 1:**
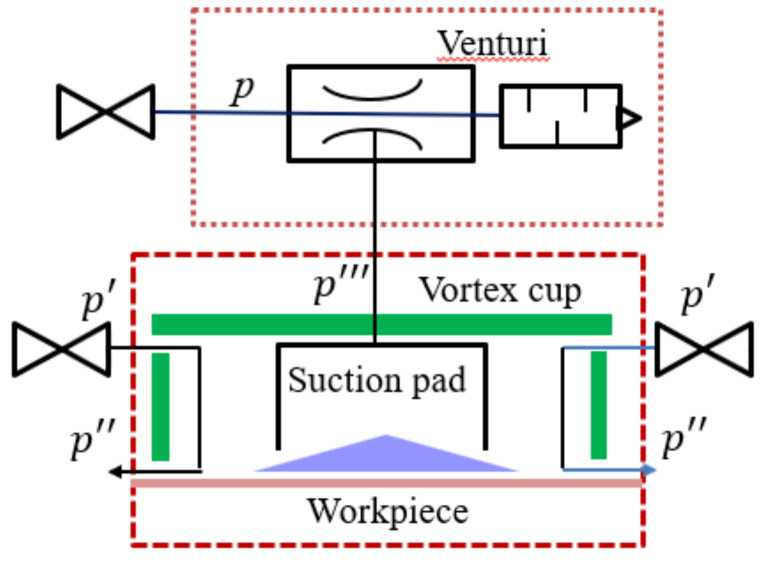
Block diagram of a combined venturi–vortex head.

**Figure 2:**
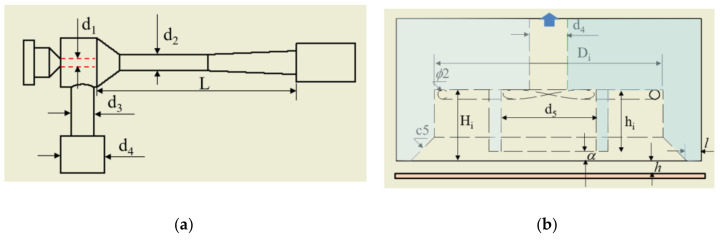
Drawing of a venturi–vortex head: (**a**) upper part; (**b**) lower part.

Our previous study [21] analyzed the effects of design factors on the vortex cup’s (i.e., the head’s) cylindrical columns. From these results, we confirmed that the presence of the hollow column controlled the instability of the vortex airflow at the center of the cup and maintained an effective vacuum. Based on these results, we propose the combination of the two units shown in Figure 1. This structure has a venturi nozzle portion on the outside of the vortex cup, and a cylindrical suction pad inside the vortex cup is connected to the venturi nozzle region. The vortex cup has two inlets on the tangential wall, which induces a vortex flow along the cup wall, creating a vacuum inside. The end of the combined head has less interference in this area because it has an air gap that protects the vacuum inside the vortex and a vacuum gap with a small mutual pressure differential environment at the bottom of the suction pad. Figure 2 is a detailed drawing with geometric elements.

However, this structure must individually manage the pressure (*p*) in order to generate the venturi-side vacuum (*p‴*) and vortex-side pressure (*p′*) to form the locally generated pressure (*p‴*) required for non-contact, as shown in Figure 1. As a result, the actual lifting force expressed in Equation (1) is determined by balancing the forces acting at a certain distance. In the force balance of Equation (1), the first and second forces of Equation (1) act on the venturi vacuum pad and in the vortex cup, respectively, and the last term is the weight (*W* (N)). The air gap (*h*, (mm)) depends on vortex outlet velocity (*v_ext,_* (m/s)) and discharge mass flow rate ($\dot{m}$, (kg/s)), as described in Equation (2). If the forced swirling flow of the wall jet is maintained in the strong vortex region $\left(r_{1}<r<r_{a}\right)$, the radial pressure gradient changes, affecting the radial and axial flows with various internal channels:

(1) $$F_{lifting}={\left(\pi r_{1}^{2}p_{vacuum\;pad}+2\pi \int_{r_{1}}^{r_{a}}p_{vortex}rdr-W\right)}_{with\;z=h}=0$$

(2) $$h=\frac{\dot{m}}{2\pi r_{a}v_{ext}\rho }$$

where ρ is the density, $r_{1}$ is the inner radius of the suction pad (*d*_5_/2), and *r_a_* is the inner radius of the head, $\left(D_{i}+c5\right)/2$, as shown in Figure 2 and Table 1.

## 3. Numerical Method

Since the functions of the vortex cup and venturi tube, which are combined modules in Figure 1, appear as physical forces on the surface of the object, the boundary elements were examined via separating the functions of the two modules. Here, the boundary condition in the open non-contact state primarily comprises numerical calculations for handling the complex relationship of the combined modules.

The compact venturi model minimized air consumption based on commercially available specifications [28]. Table 1 shows the design elements and specifications of the present venturi–vortex head. The venturi’s first nozzle diameter (*d*_1_) was 1.0 mm, the ratio of the venturi nozzle was (*d*_2_*/d*_1_) 1.4–2.4, and the venturi tube had a length of 28 mm until the mixing and expansion zone. The diameter of the passage portion from the upper venturi to the lower suction pad expanded from 3.0 mm to 8.0 mm. The vortex cup’s basic shape utilized some of the results presented in a previous study [21]. The vortex cup’s outer diameter was extended to 64 mm, which was required for inserting the venturi vacuum pad into the center of the vortex cup. As performed previously, the tip length and the cup’s inner height were set to 3 mm and 15 mm for proper swirl motion, respectively. A suction pad with a diameter of 20 mm and a height of 13 mm was placed in a vortex cup with an internal diameter of 48 mm. Two inlets were connected tangentially to the cup’s inner wall, and the tip portion had a chamfer (c5).

**Figure 3:**
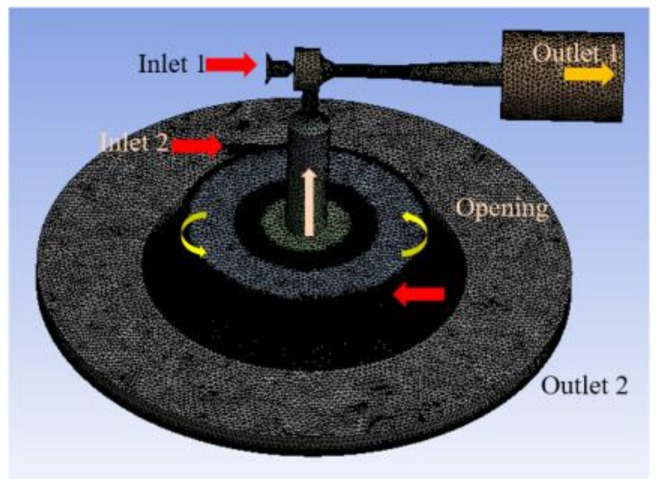
Assembled modeling of computational domains with a non-constructed grid.

The numerical simulations were conducted by using CFX code. Figure 3 shows a computational fluid area mesh with solids removed for the combined head. Each module’s discharge interface almost reached the atmospheric pressure level, preventing flow such as reverse circulation. The entire computational fluid domain was applied as a non-uniform spaced grid (tetrahedron) in order to counteract physical changes that vary from shape to shape. The continuous equation, momentum equations, and the k–ε turbulence model were applied. The flows were assumed to be steady and incompressible on the premise of using a low working pressure. The confidence level of the calculation was determined at the 4.9 million nodes by taking into account the venturi nozzle periphery and the forced vortex. The convergence condition was set to 10^−^^4^ in common with governing equations used. Table 2 lists the conditions of the present numerical simulation, including boundary conditions.

## 4. Results and Discussion

### 4.1. Vacuum Pumping by the Venturi Chamber

**Figure 4:**
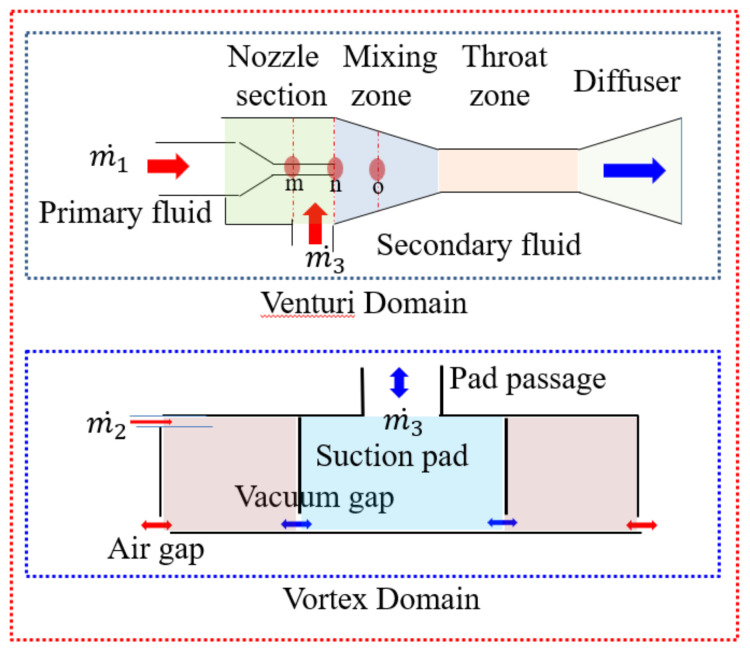
Schematic of venturi nozzle structure and an opening structure of the vortex cup–suction pad.

The venturi’s first nozzle exits were placed at positions *m*, *n*, and *o* in order to evaluate the vacuum performance of the chamber. The base alignment of the nozzle exit at *n* was the entry of the mixing chamber, as shown in the venturi domain of Figure 4. Furthermore, the vortex domain (diameter, *d*_5_ = 20 mm) with a lower gap opening was set so that the air gap (*h*) would be 0.3–1.0 mm and the vacuum gap (*α*) would be 0.0–4.0 mm while satisfying the non-contact condition described in Equation (3).

Total opening gap in the suction pad (*h* + *α*) ≥ Air gap in the vortex region (*h*) (3)

#### 4.1.1. Venturi Performance with the Closed Suction Pad

When using compressed air at 1.5–4.0 bar at 25 °C, the applied mass flow rate ranged from 6.466 × 10^−^^4^ kg/s to 1.085 × 10^−^^3^ kg/s. The higher the operating flow rate by the inlet pressure, the higher the pumping capacity in the chamber area depending on the mixing chamber’s structural shape. Among other factors, the mixing chamber associated with the second nozzle region, following the injection flow rate, had a significant difference in flow characteristics because of velocity distribution, pipe diameter, and flow rate.

**Figure 5:**
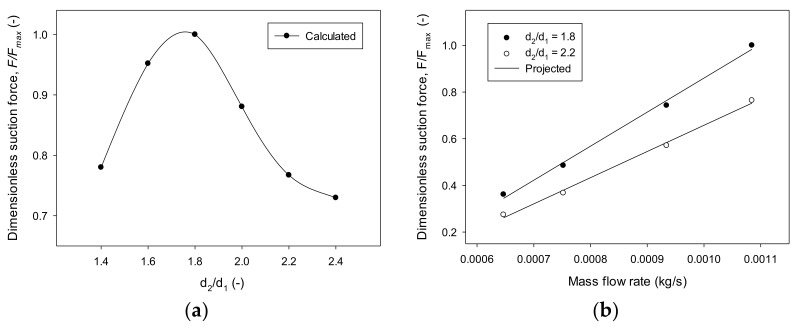
Dimensionless suction force ratio in the venturi chamber with a closed suction pad: (**a**) different nozzle ratios, *d*_2_*/d*_1_ using a mass flow rate (${\dot{m}}_{1}$) of 6.466 × 10^−^^4^ kg/s; (**b**) different mass flow rates (${\dot{m}}_{1}$).

A flow rate of 6.466 × 10^−^^4^ kg/s was applied in order to examine its effect on suction force according to the throat ratio of the nozzle constituting the mixing chamber. The first nozzle exit existed at the entry of the mixing chamber, and the nozzle diameter ratio was 1.4 to 2.4 times. The suction calculation for the variation in the nozzle diameter ratio is shown in Figure 5a. This result produced nondimensional values based on the highest suction force in the range examined. The suction force increased and decreased in a second relationship with a neck ratio of 1.8 times the nozzle diameter. Similarly, Figure 5b compares the suction force maintained in the closed suction pad (diameter, *d*_4_ = 8 mm) for each injection flow rate in a dimensionless manner with the maximum value within the application range. It showed an increasing trend proportional to the flow rate used. As flow rate increased, the suction linearly increased, and when the ratio of the nozzle diameter was 1.8, the rate of increase was higher than that of 2.2. A higher flow rate with high-pressure energy more significantly influenced flow structure, creating a vacuum in the chamber. The flow structure was affected by the surrounding shape structure constituting the chamber. This analysis offers an effective incompressible flow motion below a specific flow rate, assuming the use of low energy due to the product’s compact size. Therefore, very high speeds may appear as an undesired vacuum when calculating incompressibility using Equation (4). The limit was a vacuum pressure formed in the venturi chamber and confirmed by the limit range (<−1.0 × 10^5^ Pa).

(4) $$\rho g-\nabla p+\mu \nabla^{2}\vec{V}=0$$

**Figure 6:**
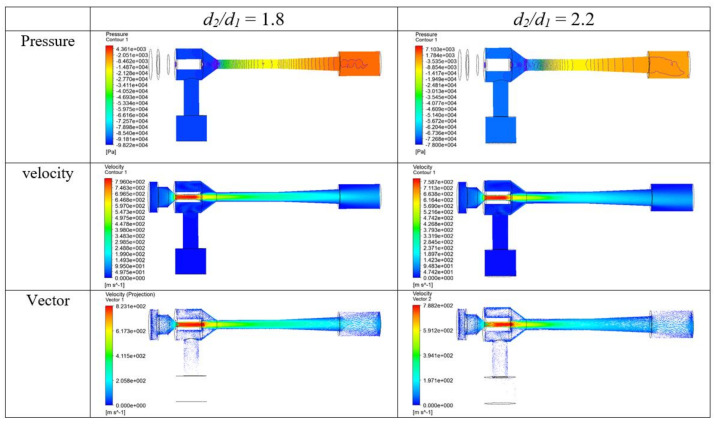
Pressure, velocity, and vector contours (*d*_2_*/d*_1_ = 1.8, 2.2 and ${\dot{m}}_{1}$ = 6.466 × 10^−^^4^ kg/s).

Critical conditions must be maintained in order to form a flow structure for high shearing action and inflow using the flow rate in the chamber space. The optimum ratio of the critical nozzle neck size depends on the effect of mutual interaction on the design factors that make up the chamber shape. As the core flow passed through the secondary nozzle, a comparative analysis was performed through pressure gradient and velocity distributions, as shown in Figure 6. Nozzle diameter ratios (*d*_2_*/d*_1_) of 1.8 and 2.2 were used. When the ratio was 1.8, a very steep pressure gradient appeared in the second nozzle. Moreover, the flow reduced the circulation at the contracting region or minimized the friction loss of the pipe wall in the second nozzle. Based on these results and on the selection of *d*_2_*/d*_1_, the length and diameter of the venturi nozzle were determined by the correlation between the appropriate gradient evaluated considering the working pressure and the discharge rate that produced the optimum condition.

**Figure 7:**
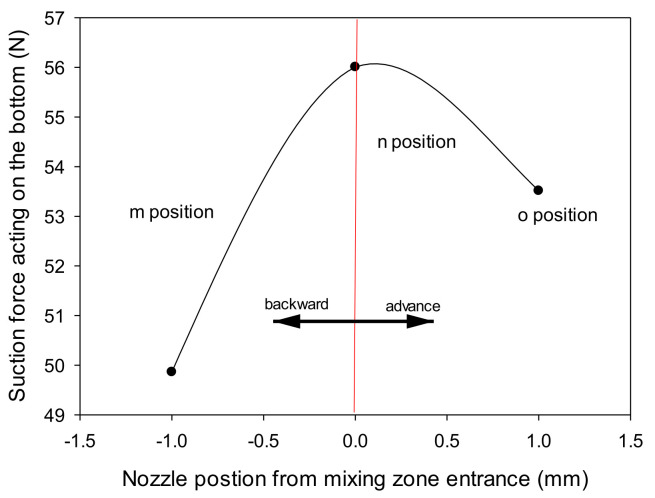
Comparison of suction force according to the exit position of the first venturi nozzle.

Figure 7 shows the change in the exit position of the first nozzle affecting the suction force. The suction pad was not taken into account because it was blocked in the pad passage. The results exhibited a different suction force when the nozzle exit was forward, backward, or aligned at the entry of the contraction zone. The sprayed fluid moved rapidly to any environment within the contraction passage along with the shearing action. The flow structure along the second nozzle played a prominent role in forming pressure in the mixing chamber.

Moreover, it affected the structure of energy transfer or flow injected and moved according to the exit distance of the first nozzle. When the first nozzle exit was aligned with the entry of the contraction zone, suction in the pad passage was at its maximum. Again, when repositioned to *m* or *o*, it was 0.89 or 0.95, respectively, compared to the maximum level (*n*).

#### 4.1.2. Sealing Effect of the Vacuum Gap in the Suction Pad

**Figure 8:**
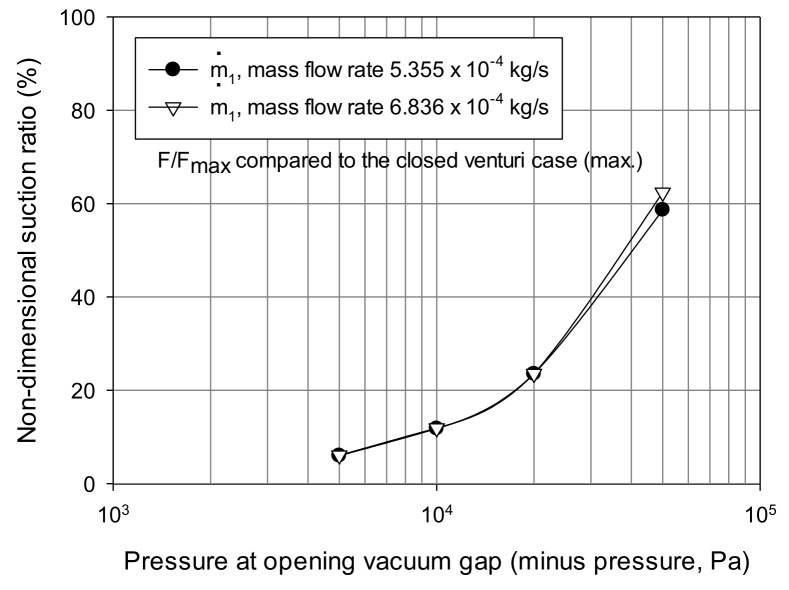
Nondimensional suction force, indicated by the opening gap pressure of the suction pad (nondimensionalized by the maximum grip force obtained at the closed type).

Figure 8 shows the grip capacity maintained at different opening negative pressure environments when the non-contact vacuum gap (*α*) between the tip of the venturi suction pad and the surface of the workpiece was set to 2.0 mm. In this case, the boundary condition of the gap was set to the closed state and various relative opening pressure ranges (−50 kPa–0 Pa). This setting was the same as that when the venturi suction pad was present inside the vortex cup. Regardless of the vortex cup region, the vacuum gap boundary was uniformly exposed by the pressure difference from the outside. These values were provided as relative pressure values at the opening boundary.

When the venturi suction pad acted without a vacuum gap, the maximum suction capacity was −26.79 N for a mass flow rate (${\dot{m}}_{1}$) of 6.836 × 10^−4^ kg/s and pad passage size of $\pi /4{\left(20\right)}^{2}$ mm^2^. When non-contact gaps existed, the gap boundary was set to relative open pressure considering the vacuum level generated within the vortex cup. The difference in vacuum level generated by the vacuum pad caused an inflow along the boundary. With different non-contact opening conditions, each calculated suction force was nondimensionalized by the maximum grip force of the closed case. They showed the resultant grip capacity acting on the workpiece surface while maintaining equilibrium through this gap in proportion to the relative pressure. When the pressure near the venturi’s pad tip reached the atmospheric pressure level, we found that the vacuum could not be collected even in the 2.5 mm gap (*h* + *α*).

The difference between the external vacuum environment created in the vortex cup and the vacuum in the venturi pad must be decreased in a non-contact open environment by reducing the amount of airflow rate through the opening boundary of the vacuum gap. Therefore, the proper combination of the two modules offers a mutual sealing effect and can be managed individually.

### 4.2. Vacuum Generation of the Vortex Cup

The air gap (*h*) was in the range of 0.3–0.9 mm for evaluating changes in the grip force. The vacuum gap (*α*) of the venturi suction pad was set to 2 mm thickness in the vortex domain without the suction pad passage shown in Figure 4. The vortex inlet pressure used in this case was 4 bar, and the corresponding air mass flow rate was 2.83 × 10^−^^3^ kg/s for each inlet port.

#### 4.2.1. Effect of the Air Gap and the Vacuum Gap in the Vortex Cup

Vortex cups were made so that the main flow rotates near the cup wall formed, and various circulation flows occurred around the main flow interface. A steep pressure gradient was inwardly formed from the mainstream boundary layer, and the air flowed in through the shearing action to interior of the boundary layer. Through this continuous process, the center of the vortex proceeded to a low-vacuum area. In addition, the proper flow-inducing space inside a simple vortex cup caused a stable flow in the vortex. According to previous studies, a cylindrical column was added to the center of the vortex cup in order to control the unstable flow of the spiral while maintaining a smooth swirling flow and stable grip [3,21]. Similarly, in the model of their studies, the suction pad functioned as a space leading to flow control in the vortex cup. More importantly, this space must have a suitable vacuum range to provide a balanced vacuum sealing effect to the free surface of the suction pad.

**Figure 9:**
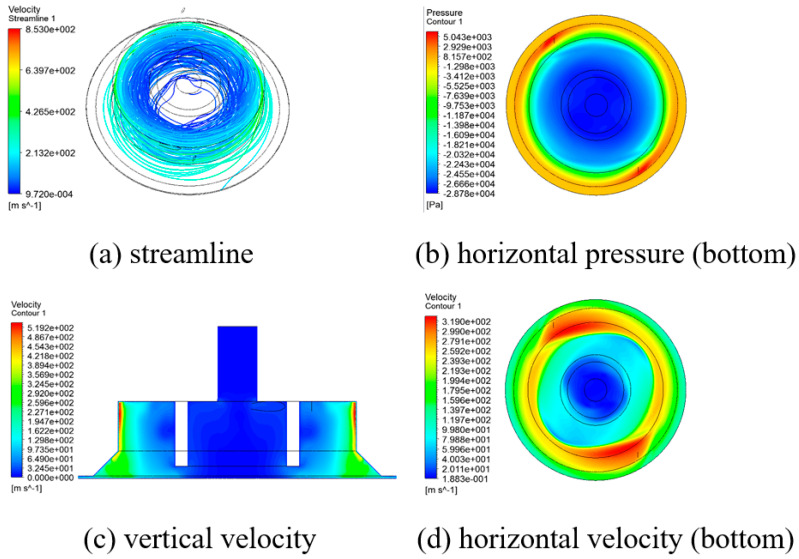
Flow behavior by the vortex cup; the air gap was 0.5 mm, and the vacuum gap was 2.0 mm.

**Figure 10:**
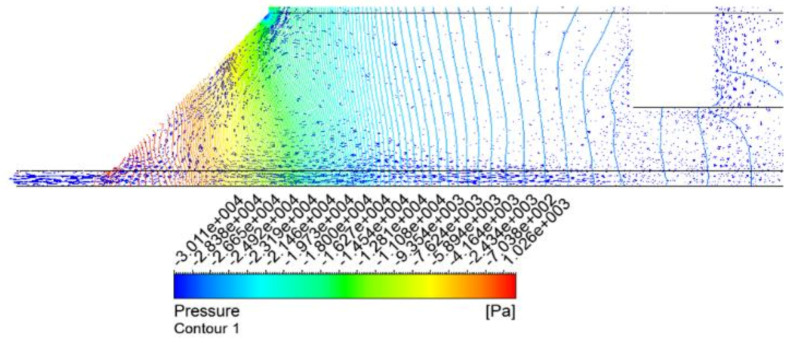
Pressure contour with vector field and the enlarged image at the exit and bottom areas of Figure 9c.

Figure 9 showed the streamline, pressure, and velocity distributions in the vortex cup when an air gap of 0.5 mm was applied. These results were obtained by using a simple cup with no tip size, a chamfer size of 5 mm, and an inner height of 15 mm. While rapidly rotating spirally in the tangential direction around the wall, as shown in Figure 9a, the main flow introduced air from outside the boundary layer and formed a vacuum around and inside the suction pad. The pressure gradient that formed on the workpiece surface in Figure 9b appeared steep at the main flow boundary. This result was significantly dependent on the chamfer shape associated with the discharge flow. The velocity profile of the vertical cross section is shown in Figure 9c. The detailed pattern is shown in Figure 10 with velocity vectors along with pressure distributions. The majority of the main stream with radial and axial movements was released into the air gap, and some circulated inward. Proper circulation and rapid pressure gradient patterns around the air gap indicate vacuum generation inside the cup. Unstable flow behavior appears on the workpiece surface of Figure 9d. The overlapping flow at the nozzle exit of the asymmetrical structure can usually be solved by the optimum suction pad space or by modifying it to the appropriate cup specifications [15].

**Figure 11:**
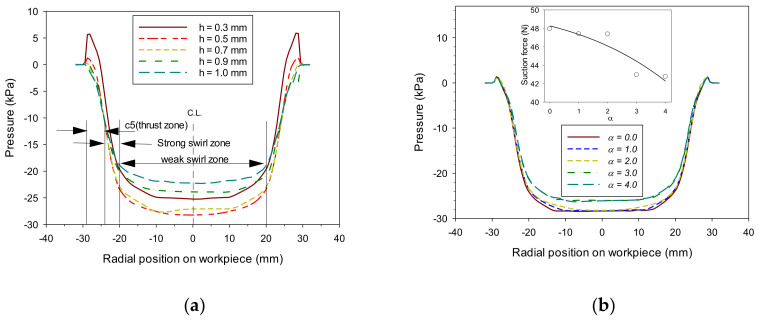
Pressure distribution acting on the surface of the workpiece formed by the vortex cup: (**a**) effect of air gaps (*h*) at 2 mm vacuum gap (*α*); (**b**) effect of vacuum gaps (*α*) at 0.5 mm air gap (*h*).

Figure 11a shows the vacuum variation acting on the workpiece surface in the radial direction when the vacuum gap (*α*) was 2.0 mm, and the air gap (*h*) changed from 0.3 to 1.0 mm. Figure 11b shows a similar shape when the air gap changed to 0.5 mm, and the vacuum gap changed from 0 to 4 mm. The smaller the air gap, the more complicated the discharge environment, as shown in Figure 9. The circulation between the gap and the surrounding layer becomes greater, which affects vacuum development. When the air gap was 1.0 mm, the region around the chamfer (±(20–28) mm in the radial direction) with the fast circulation’s inflow effect slightly affected internal vacuum generation.

When the vacuum gap increased to 3 mm, a meaningful change in internal pressure (or suction force) was observed, as shown in Figure 11b. The column in the vortex cup affects internal flow depending on its shape, size, and position, and this resulted in a change in the internal vacuum. In the case of a vacuum gap of 2 mm or more under the current column conditions, the effect of suppressing the internal inflow was lost, which resulted in nearly identical results.

**Figure 12:**
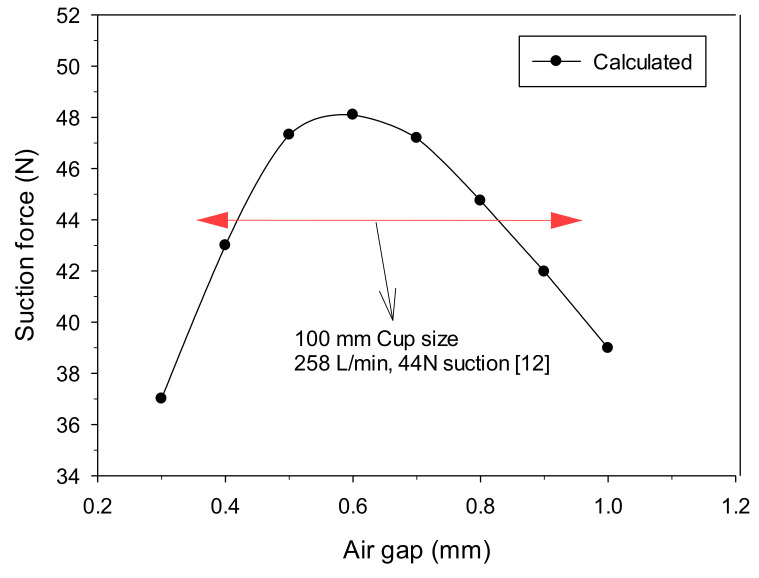
Vortex cup performance with different air gap (*h*) openings. The vortex area was connected to a closed suction pad region with a vacuum gap (*α*) of 2.0 mm.

Conversely, the smaller the vacuum gap in Figure 11b is, the smaller the pressure formed inside the suction pad. Overall, this pressure was not a significant difference but seemed to be the small effect of circulating flow formed under the suction pad. Considering the convex up curved surface occurring when thin plates were gripped, maintaining the thickness of the vacuum gap to be larger than that of the air gap is crucial. For this reason, it was a vacuum gap (up to 2 mm) above a certain level, and by allowing the vacuum created in the vortex cup to equilibrate without a notable difference, the vacuum effect created by the venturi was sufficiently maintained. The vortex cup’s ability to grip the workpiece was calculated from −48.1 to −37 N, as shown in Figure 12. Since this value can protect the venturi’s relatively weak vacuum level, a sealing effect can at least be expected through the vacuum gap, and the most efficient air gap was found at 0.6 mm.

#### 4.2.2. Influence of the Tip Length of the Vortex Cup

The air gap size can be observed as a simple relationship between the discharge air amount and the average speed for pushing out the main flow based on Equation (2). However, this resulted from the flow in the balancing process against the thrust of the main flow exiting through the air gap. It was important to control the flow circulating internally with the proper shape and operating conditions of the end.

**Figure 13:**
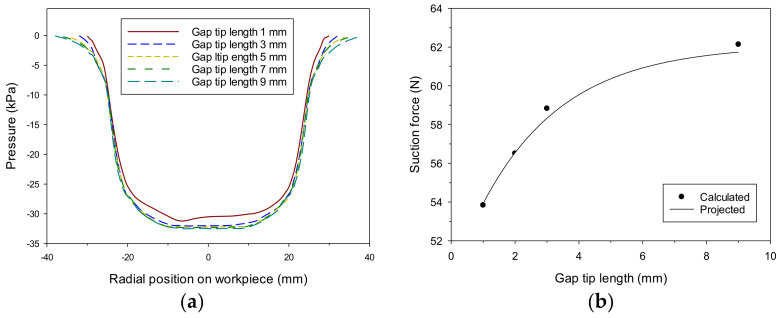
(**a**) Pressure distribution acting on the surface of the workpiece built by the vortex cup; different gap lengths (*l*) and 0.6 mm air gap; (**b**) vortex cup performance with different gap tip lengths (*l*), air gap (0.6 mm), and a vacuum gap (2.0 mm).

Figure 13a shows the pressure distribution formed inside the cup according to the tip length of the gap. When the air gap was 0.6 mm and the tip length of the gap varied from 1 mm to 9 mm, slight differences appeared in the vacuum variation by influencing internal circulation. No notable change was obtained, except for the tip length of 1 mm. As the tip length increased, the possibility of causing circulation internally and the intensity increased, contributing to vacuum formation to a certain extent. The change in the tip length greatly affected the formation of the gap in force balance. If there were no or extremely small tip lengths, a small gap could have been formed. Figure 13b shows the ability to hold a workpiece under the same conditions. For models with a gap of 5 mm and neglecting the tip length, it was calculated as −48 N, as shown in Figure 12. By comparison, increasing the length of the air gap tip under a constant vacuum gap correspondingly increased grip force. However, as described in the previous section, the actual air gap varies in the process of balancing forces. The longer gap tip increased instability at the end of the head, whereas the shorter gap tip decreased the thickness of the gap. Smaller gap tips are advantageous for holding small and robust workpieces. If the gap tip needs to be significant, it is suitable for holding large and flexible plates.

### 4.3. Grip Performance of the Combined Venturi–Vortex Gripper

Two modules were combined by applying large specifications of the vortex cup identified in previous studies [3,7,21], and the findings [21], which involved a venturi nozzle ratio *d*_2_*/d*_1_ ≤ 2.2, an air gap of 0.5 mm, and a vacuum gap of 2.0 mm, are shown in the venture–vortex domain of Figure 4. When using two modules in combination, the grip performance was evaluated by increasing the working pressure by 0.5 bar in the range of 1.5–4.0 bar.

**Figure 14:**
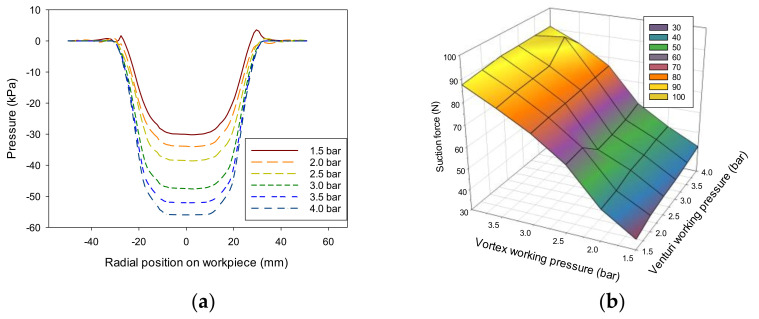
Functional performance of combined venturi–vortex heads at different working pressures (flow rates): (**a**) pressure distribution (using different vortex cup working pressures and 3.5 bar venturi inlet port); (**b**) mesh of suction force compared to the maximum suction force at a working pressure of 4 bar.

Figure 14a shows the variation in the pressure distribution formed on the workpiece by different working pressure levels in the combined method. When the venturi used a working pressure of 3.5 bar and the working pressure range of the vortex cup was 1.5–4.0 bar, the total air consumption of the vortex cup was 3.49 × 10^−3^ to 5.74 × 10^−3^ kg/s. Vortex cups with high air consumption more significantly affected vacuum formation than the venturi; thus, pressure distribution variation was investigated for vacuum levels at the bottom of the combined module. As previously discussed for each module, this variation was expressed as the nondimensional suction capacity shown in Figure 14b. A performance curve showed the effect of reduced air consumption on grip capacity due to the change in working pressure applied to each module in the range of 1.5–4.0 (or 3.5) bar.

Two modules showed a significant difference in air consumption. However, the maximum (4 bar) and minimum (1.5 bar) working pressures differed by 2.67 times with the gripping level. The actual air gap was formed depending on many factors, but the air gap resulted from setting the same value to 0.5 mm for the analysis. The change in grip force from the contour plot was due to the vortex cup’s air consumption. Overall, the change in the venturi operating pressure with low air consumption was not significantly different from the change in the suction force of the vortex cup. Therefore, using the lower operating pressure of the vortex cup or further reducing its size would be feasible. In addition, venturi’s features can be expanded or increased depending on air consumption in a parallel or series configuration of modules.

**Figure 15:**
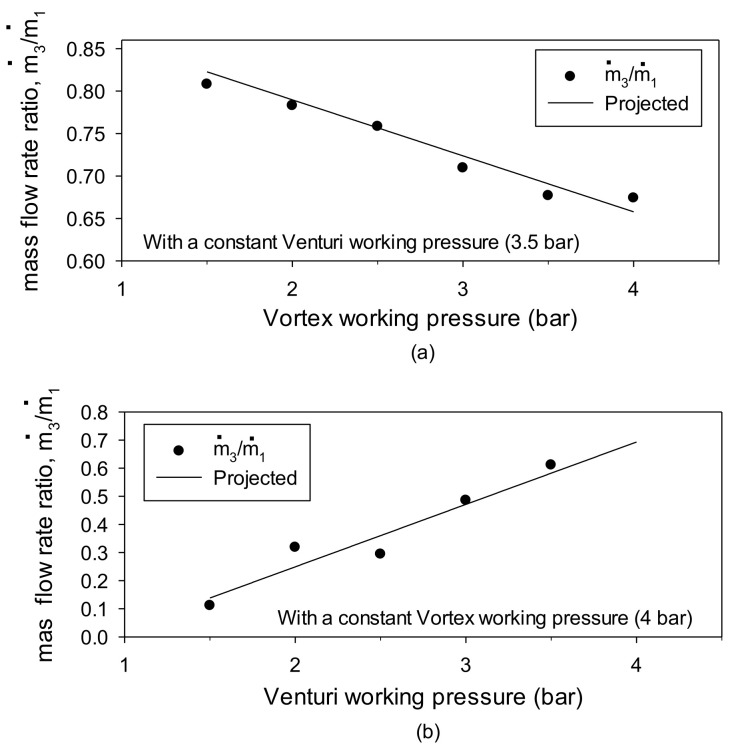
Venturi suction flow rate versus input flow rate when using different working pressures in the assembled device: (**a**) vortex work; (**b**) venturi work.

Figure 15 shows the efficiency as the ratio of the airflow rate through the venturi suction pad based on the amount of compressed air used for each module in the combined module. The first was provided with various working pressures on the vortex module, and a fixed working pressure of 3.5 bar was assigned to the venturi. The open interface environment between the combined modules was demonstrated by a vacuum balance between the central region of the vortex cup and the venturi pad. Conversely, applying working pressures only changed each module. By comparing the air inflow ratio (${\dot{m}}_{3}/{\dot{m}}_{1}$) flowing from the venturi pad area to the venturi chamber, we found that a higher operating pressure of the vortex cup resulted in better sealing of the open interface on the pad side and less inflow. A higher vacuum resulted in a lower inflow rate to the venturi chamber. This effect is a percentage of the flow rate through the venturi suction pad.

Conversely, the second fixed the vortex working pressure at 4 bar and increased the venturi’s working pressure, increasing the inflow ratio due to the vacuum pressure increase in the venturi chamber. These variations demonstrate a linear relationship, and the operation of the vortex cup had a much more significant effect in assisting the venturi’s ability. The combined head contained some of the venturi functions in the vortex cup, and the air consumption required for the vortex flow was functionally required over a certain amount, as shown in Figure 14b.

Therefore, as a result of comparing the mass flow rate ratio in Figure 14, linear relationships of y = −0.198 x_1_ − 0.922 (based on the vortex operating pressure) and y = 0.222 x_2_ − 0.195 were obtained based on the venturi’s operating pressure. Here, y is the mass flow rate ratio, x_1_ is the venturi work pressure, and x_2_ is the vortex work pressure.

## 5. Conclusions

We numerically analyzed grip capacity when the venturi module was combined with the vortex cup. The combined head had the structure of a separated vacuum pad (connected to the external venturi) inside the vortex cup. We found that the head maintained the vacuum capacity inside each volume in a reliable manner. For a non-contact gripping environment, this was achieved by controlling the airflow through the air gap and vacuum pad gaps in the internal structure of the combined head.

Furthermore, design effects were analyzed in terms of the vacuum pumping flow around the small-sized venturi and the swirl flow of the vortex cup in order to confirm their behaviors. For the applied head size (inner diameter: 40 mm), a stable grip was achieved by applying an air gap of 0.5 mm, air gap lengths of 3 mm, vacuum gaps of less than 2 mm, and venturi tube specifications that satisfy *d*_2_*/d*_1_ ≤ 2.2.

The combined effect with the vortex cup exhibited a theoretical vacuum gripping force of up to double the level it was before and contributed to maintaining additional grip forces of the venturi vacuum pad in a non-contact environment. The vacuum pad (cylinder type) helped control a swirl flow in the vortex cup and sucked up the air with an external venturi. The combined head is worth the application towards large flat objects with double-up grips, various usage options, and multiple arrangements.

## Figures and Tables

**Table 1:** Design parameters and specifications of the venturi–vortex airhead.

Design Parameter (Symbol)	Value (Unit, mm)
Venturi nozzle diameter (*d*_1_)	1
Constant section diameter (*d*_2_)	1.4–2.4
Upper vacuum intake port (*d*_3_)	3
Lower vacuum intake port (*d*_4_)	8
Suction/constant/diffuser length (*L*)	28
Cup tip length (*l*)	0–9
Cup inlet diameter (*a*)	2
Suction pad height (*h_i_*)	13
Inner vortex cup diameter (*D_i_*)	48
Suction pad diameter (*d*_5_)	20
Inner vortex cup height (*H_i_*)	15

**Table 2:** Conditions of the present numerical simulation.

Parameters	Conditions
Fluid domain	Steady state, incompressible
Working fluid	25 °C air
Turbulence model	k–ε model (intensity 5%)
Inlet	venturi inlet 1 (mass flow rate)
	vortex inlet 2 (mass flow rate)
Outlet	pressure outlet (atmosphere)
Opening	Relative pressure
	Venturi only (0 Pa below)
	Vortex (0 Pa)

## Data Availability

Not applicable.
